# The *Moraxella catarrhalis* phase-variable DNA methyltransferase ModM3 is an epigenetic regulator that affects bacterial survival in an in vivo model of otitis media

**DOI:** 10.1186/s12866-019-1660-y

**Published:** 2019-12-09

**Authors:** Luke V. Blakeway, Aimee Tan, Joseph A. Jurcisek, Lauren O. Bakaletz, John M. Atack, Ian R. Peak, Kate L. Seib

**Affiliations:** 1 Institute for Glycomics, Griffith University, Gold Coast, Queensland 4215 Australia; 2 Center for Microbial Pathogenesis, The Research Institute at Nationwide Children’s Hospital, Columbus, OH 43215 USA; 3 School of Medical Science, Griffith University, Gold Coast, Queensland 4215 Australia

**Keywords:** *Moraxella catarrhalis*, Restriction-modification systems, Phase variation, Phasevarion, Methylome analysis, Epigenetic regulation

## Abstract

**Background:**

*Moraxella catarrhalis* is a leading cause of otitis media (OM) and chronic obstructive pulmonary disease (COPD). *M. catarrhalis* contains a Type III DNA adenine methyltransferase (ModM) that is phase-variably expressed (i.e., its expression is subject to random, reversible ON/OFF switching). ModM has six target recognition domain alleles (*modM1–6*), and we have previously shown that *modM2* is the predominant allele, while *modM3* is associated with OM. Phase-variable DNA methyltransferases mediate epigenetic regulation and modulate pathogenesis in several bacteria. ModM2 of *M. catarrhalis* regulates the expression of a phasevarion containing genes important for colonization and infection. Here we describe the phase-variable expression of *modM3*, the ModM3 methylation site and the suite of genes regulated within the ModM3 phasevarion.

**Results:**

Phase-variable expression of *modM3*, mediated by variation in length of a 5′-(CAAC)_n_-3′ tetranucleotide repeat tract in the open reading frame was demonstrated in *M. catarrhalis* strain CCRI-195ME with GeneScan fragment length analysis and western immunoblot. We determined that ModM3 is an active N6-adenine methyltransferase that methylates the sequence 5′-AC^m6^ATC-3′. Methylation was detected at all 4446 5′-ACATC-3′ sites in the genome when ModM3 is expressed. RNASeq analysis identified 31 genes that are differentially expressed between *modM3* ON and OFF variants, including five genes that are involved in the response to oxidative and nitrosative stress, with potential roles in biofilm formation and survival in anaerobic environments. An in vivo chinchilla (*Chinchilla lanigera)* model of otitis media demonstrated that transbullar challenge with the *modM3* OFF variant resulted in an increased middle ear bacterial load compared to a *modM3* ON variant. In addition, co-infection experiments with NTHi and *M. catarrhalis modM3* ON or *modM3* OFF variants revealed that phase variation of *modM3* altered survival of NTHi in the middle ear during early and late stage infection.

**Conclusions:**

Phase variation of ModM3 epigenetically regulates the expression of a phasevarion containing multiple genes that are potentially important in the progression of otitis media.

## Background

*Moraxella catarrhalis* is a human respiratory tract pathogen that is often carried asymptomatically in the nasopharynx [1], but frequently causes otitis media (OM) in infants and children, and exacerbations of chronic obstructive pulmonary disease (COPD) in adults. Along with *Streptococcus pneumoniae* and non-typeable *Haemophilus influenzae* (NTHi), *M. catarrhalis* is among the most prevalent bacterial causes of OM where it is detected by PCR in up to 56% of middle ear fluid associated with OM [2]. *M. catarrhalis* also causes approximately ~ 10% of exacerbations of COPD each year in the USA [3]. *M. catarrhalis* is also important as a co-pathogen in OM with *S. pneumoniae* and NTHi, as noted in both observational [4] and experimental studies [5, 6]. There is currently no vaccine available to prevent *M. catarrhalis*-mediated disease, and increased research is needed to identify stably-expressed vaccine antigens, correlates of protection, and to understand the progression from asymptomatic carriage to symptomatic disease.

Phase variation is the high frequency, reversible, random ON/OFF or graded switching of gene expression [7, 8]. Phase-variable gene expression is an important aspect of bacterial pathogenesis that aids in adaptation to changing host microenvironments, and which can aid immune evasion which has implications for vaccine development [9]. Several *M. catarrhalis* virulence factors and potential vaccine antigens, such as the outer membrane proteins UspA1 [10], UspA2 [11], UpsA2H [12] and Mid/Hag [13] (reviewed in [14]) exhibit phase-variable expression. Although phase variation is typically associated with genes encoding cell surface structures, phase variation of cytoplasmically located Restriction-Modification (R-M) systems has been observed in numerous host-adapted bacterial pathogens, as recently reviewed [15]. Phase-variable ON/OFF switching of DNA methyltransferase activity results in the presence or absence of methylation at a specific target sequence, leading to co-ordinated, epigenetically-regulated switching of expression of multiple genes across the genome. The suite of genes thus regulated are known as a phasevarion (phase-variable regulon) [15, 16]. Previously characterized phasevarions contain genes important for infection of the human host and potential vaccine candidates; for example *lbpA* and *lbpB* (encoding the lactoferrin binding proteins A and B, respectively) are regulated within the ModA11 phasevarion in *Neisseria meningitidis* [17], and the outer-membrane protein encoding gene *hopG* is regulated within the ModH5 phasevarion in *Helicobacter pylori* [18]. Switching of phasevarion expression has also been shown to modulate diverse phenotypes associated with virulence, such as biofilm formation [17], resistance to antimicrobial agents [19], resistance to oxidative stress [20], and survival within experimental models of infection [21].

We previously identified three phase-variable Type III DNA methyltransferases (*modM*, *modN*, and *modO*) that are variably distributed among *M. catarrhalis* isolates and strongly associated with phylogenetic lineage [22–24]. *modM* is the only phase-variable methyltransferase found in the disease-associated RB1 lineage and occurs in 76% of isolates from geographically and clinically diverse backgrounds [22]. Phase variation of *modM* is mediated at the translational level by a 5′-(CAAC)_n_-3′ tetranucleotide repeat tract present within its open reading frame (ORF) [23]. Analysis of the genomes of 51 *M. catarrhalis* strains identified six *modM* alleles (*modM1–6*) that vary in their target recognition domains [22]. Allelic variants of phase-variable Type III methyltransferases containing distinct target recognition domains methylate distinct target sequences, and regulate different suites of genes [17, 21]. In *M. catarrhalis*, only the most commonly occurring *modM* allele, *modM2*, has been investigated to date. ModM2 methylates the target sequence 5′-GAR^m6^AC-3′, and ON/OFF switching of ModM2 results in the differential regulation of 34 genes, including genes involved in colonisation and protection against host defences [23]. *modM3* is the second most frequently occurring *modM* allele in all strains analysed, and the most frequently occurring allele in strains belonging to the minor RB2/3 lineage [22]. Despite the RB2/3 lineage being less commonly associated with disease than the RB1 linage in several studies [reviewed in 14], the *modM3* allele is also overrepresented in middle ear isolates from children with OM [22, 23]. Here we characterise the phase-variable expression of *modM3*, the ModM3 DNA methylation site and its distribution in the genome, the genes regulated in the ModM3 phasevarion, and phenotypes of the ModM3 ON versus ModM3 OFF variants.

## Results

### *modM3* exhibits phase-variable expression

**Fig. 1:**
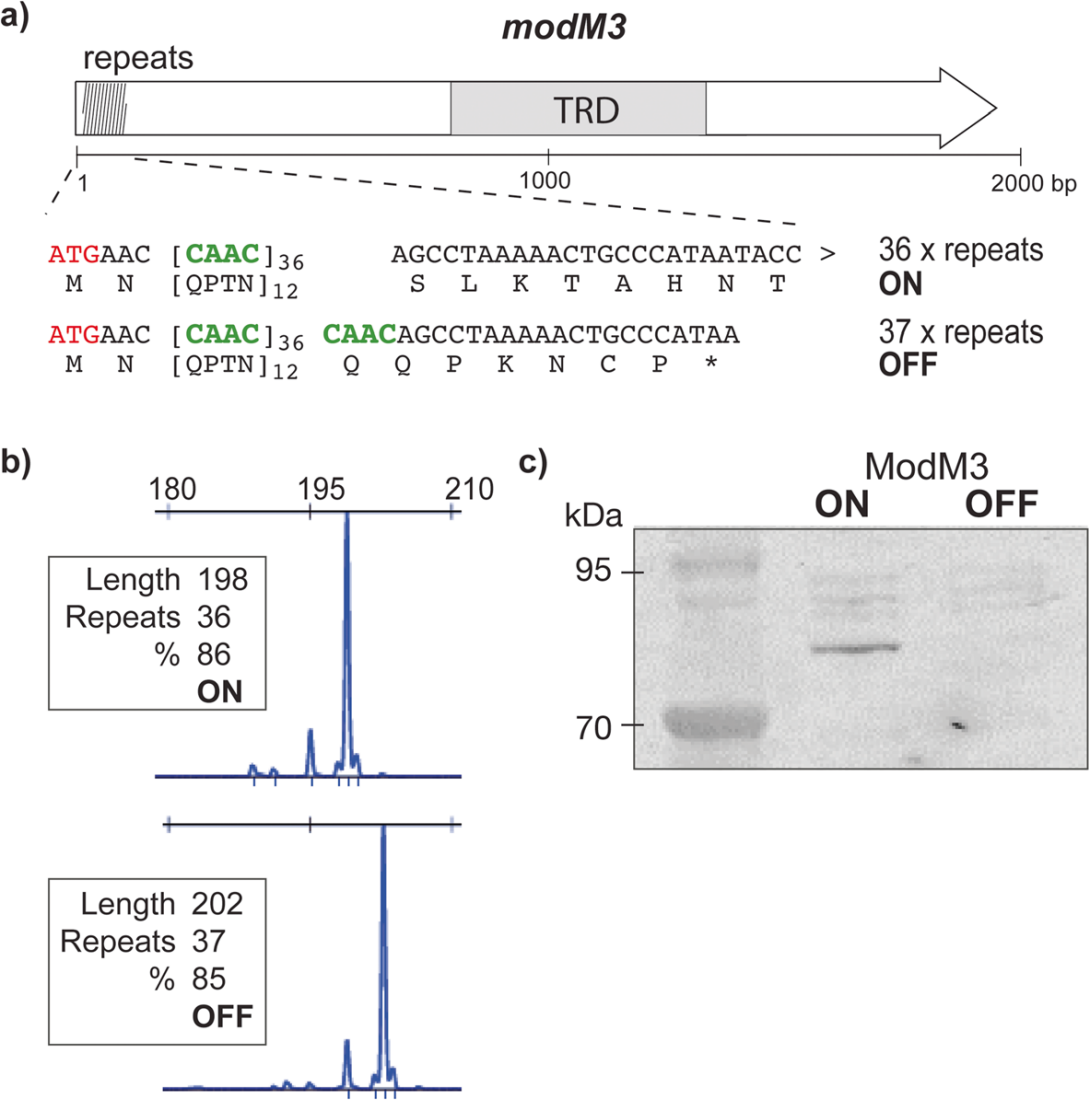
Phase-variable expression of *modM3*. **a** Schematic representation of the *modM3* gene indicating the location of the 5′-(CAAC)_n_-3′ tetranucleotide repeat region and the central target recognition domain. Translation of the full length ModM3 is possible when the repeat tract contains 36 (ON) repeat units, but not when the tract contains 37 (OFF) repeat units (an asterisk indicates a premature stop codon). **b** GeneScan fragment length analysis of the of *modM3* 5′-(CAAC)_n_-3′ tetranucleotide repeat region. The area under each fluorescent peak is proportional to the percentage of a repeat length in the population. Separate populations were isolated containing ≥85% of 36 or 37 repeats. **c)** Western blot analysis confirmed ModM3 expression in 195ME ModM3 ON (36 repeats) and the absence of expression in the 195ME ModM3 OFF sample (37 repeats)

The *modM3* gene contains a 5′-(CAAC)_n_-3′ tetranucleotide repeat tract in the N-terminal of its open-reading frame (ORF) (Fig. 1a). Different strains display varying numbers of *modM3* repeat units (e.g. 24, 31, and 35 repeats are found in strains F24, BC1 and CCRI-195ME, respectively), which is suggestive of phase variation. The *modM3* coding sequence downstream of the repeat tract is predicted to be in frame with the ATG start codon when 36 repeats are present, while a frameshift mutation resulting in a premature stop codon is predicted to occur when 37 repeats are present (Fig. 1a). To confirm *modM3* is phase-variable, single colonies of *M. catarrhalis* strain CCRI-195ME (hereafter 195ME) were screened with GeneScan fragment length analysis to measure the length of the 5′-(CAAC)_n_-3′ repeat tract and to quantify the proportion of each repeat tract length present. From an initial mixed population with repeat tract lengths ranging from 34 to 37 repeats, separate colonies containing a majority (> 85%) of 36 or 37 repeats were isolated (Fig. 1b). Western immunoblot analysis of ModM3 protein expression confirmed that expression is correlated with the number of repeat units in the 5′-(CAAC)_n_-3′ repeat tract: ModM3 is expressed (ON) when 36 repeats units are present, whereas ModM3 expression is undetectable (OFF) when 37 repeats are present, confirming that ModM3 expression is phase-variable (Fig. 1c).

### ModM3 is an active N6-adenine DNA methyltransferase that methylates 5′-AC^m6^ATC-3′

To determine the methylation specificity of ModM3, genomic DNA from our *M. catarrhalis* strain 195ME *modM3* ON and *modM3* OFF variants, as well as our *∆modM3* knockout mutant strain (generated by insertion of a kanamycin resistance cassette into the ORF of the gene), were subjected to Single-Molecule, Real-Time (SMRT) sequencing and methylome analysis. The closed genome sequence of 195ME was reported and deposited in GenBank at the time of sequencing (GenBank accession NZ_CP018059.1) [25]. Three methylated motifs were identified: one containing N6-adenine methylation and two containing 5-cytosine methylation (Table 1). N6-adenine methylation is present at the second adenine residue in the sequence 5′-AC^m6^ATC-3′ in the *modM3* ON strain but not in the *modM3* OFF or the *∆modM3* strains, indicating that this is the motif recognized and methylated by ModM3. The 195ME genome contains 4446 5′-ACATC-3′ sites in total, with 5′-AC^m6^ATC-3′ methylation observed at 100% of these sites in the *modM3* ON variants, but at 0% of these sites in the *modM3* OFF or ∆*modM3* strains (Table 1).

**Table 1:** SMRT methylome analysis of 195ME *modM3* on, *modM3* off and *∆modM3*

Methyltransferase specificity	Modified base	% of sites in genome methylated			Assignment^a^
		*modM3* ON	*modM3* OFF	∆*modM3*	
5′-AC**A**TC-3′	m6A	100	0	0	M.Mca195I
5′-GCGCGC-3′	m5C	NC	NC	NC	M.Mca195ORF6035P
5′-GCYGC-3′	m5C	NC	NC	NC	M.Mca195ORF8410P

^a^Assignment of methyltransferases to a methyltransferase specificity sequence is based on experimental evidence for M.Mca195I, and homology for M.Mca195ORF6035P and M.Mca195ORF8410P. M.Mca195ORF6035P shares 63% amino acid identity with M.HinP1I that recognizes the sequence 5′-GCGC-3′, and M.Mca195ORF8410P shares 71% amino acid identity with M.CocII that recognizes 5′-GCNGC-3′. Due to the low kinetic signal of 5mC methylation, the percentage of methylated sites are not calculated (NC)

**Fig. 2:**
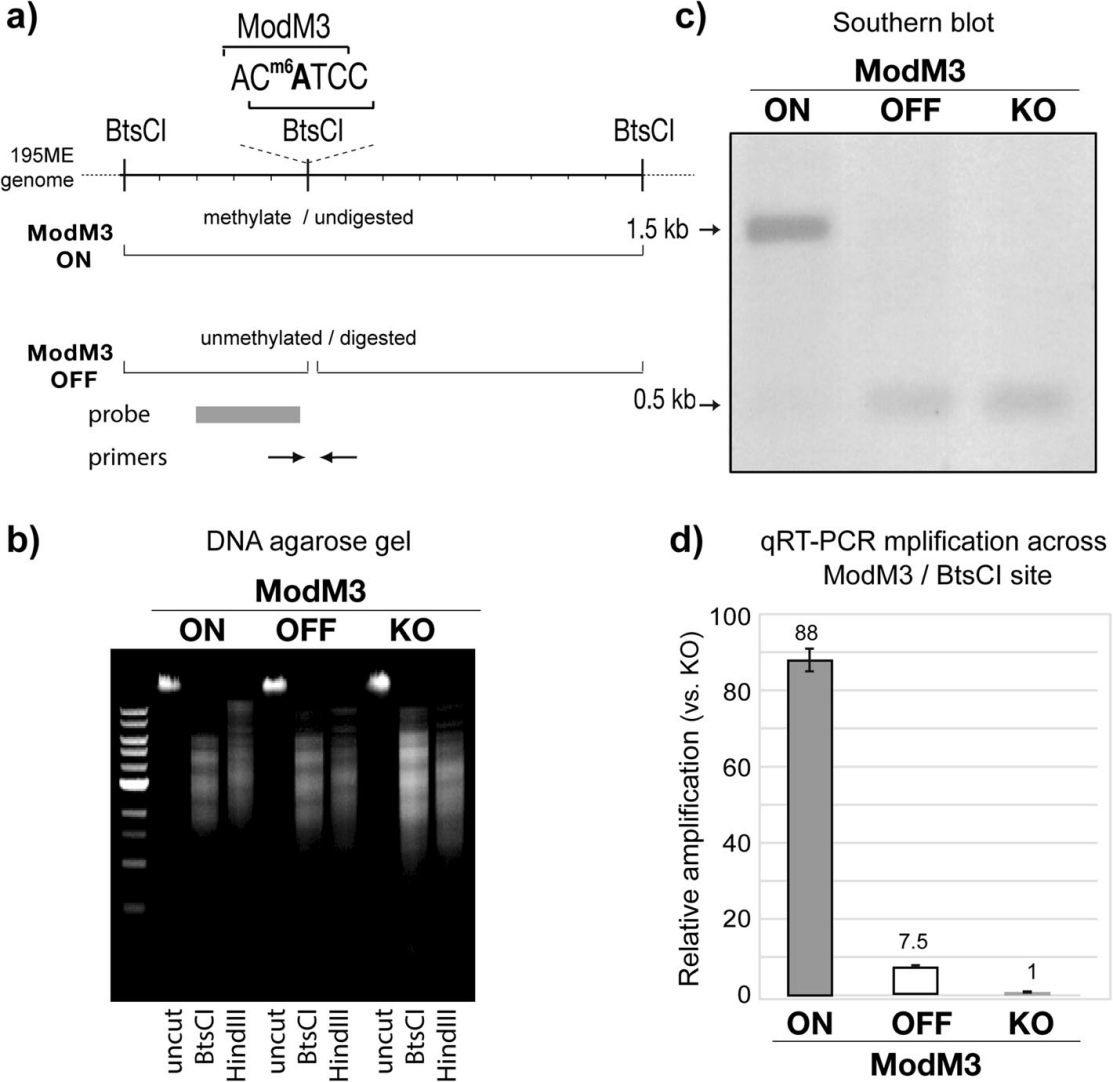
Southern blotting confirms the ModM3 methylation target sequence 5′-AC^m6^ATC-3′. **a** Schematic representation of the restriction-inhibition assay used to confirm the ModM3 methylation site. The location of the Southern blot probe, and the BtsCI and ModM3 recognition sites are shown. The central BtsCI site overlaps the ModM3 recognition sequence and is sensitive to overlapping N6-methyladenine methylation. **b** Restriction-inhibition assay of *modM3* ON, *modM3* OFF and ∆*modM3* genomic DNA using the methyl-sensitive restriction endonuclease BtsCI. The restriction endonuclease HindIII is not sensitive to methylation and is included as a control for digestion. **c** Southern blot of BtsCI digested genomic DNA isolated from *modM3* ON, *modM3* OFF, and ∆*modM3* strains. Methylated DNA in the ModM3 ON strain is protected from BtsCI digestion resulting in a 1.5 kb band. All BtsCI sites are cleaved in the *modM3* OFF and ∆*modM3* strains as ModM3 methylation is absent, resulting in a 0.5 kb band. **d** qRT-PCR indicating the relative abundance of methylated, undigested genomic DNA in *modM3* ON and *modM3* OFF relative to ∆*modM3* following digestion with BtsCI (Ct values of 18.32, 21.97, 24.77, respectively, normalised to *copB* reference)

In order to confirm the ModM3 methylation site, a restriction-inhibition assay was performed using the commercially available, methyl-sensitive restriction endonuclease BtsCI (5′-CATCC-3′) which partially overlaps the ModM3 recognition sequence (5′-AC^m6^ATC-5′) (Fig. 2a, b). Of the 2,491 BtsCI cleavage sites in the 195ME genome, 504 overlap a ModM3 recognition site and are sensitive to ModM3 methylation. There is no difference in the overall DNA digestion pattern on the DNA gel due to the majority (1,987) of BtsCI cleavage sites being cleaved in all samples. However, Southern blot analysis using a probe located adjacent to an overlapping BtsCI and ModM3 recognition sequence (and between two BtsCI sites that do not overlap with a ModM3 site; Fig. 2a) showed that genomic DNA isolated from *modM3* ON was protected from digestion with BtsCI at the site investigated with an overlapping ModM3 site, whereas *modM3* OFF and ∆*modM3* genomic DNA was cleaved (Fig. 2c), validating that ModM3 is an active N6-methyladenine methyltransferase that methylates the target sequence 5′-AC^m6^ATC-3′. A quantitative real time PCR (qRT-PCR) based assay was designed to allow rapid, sensitive quantification of the relative frequency of methylation at a specific DNA site. Primers were designed to amplify a 99 bp amplicon spanning the overlapping BstCI and ModM3 recognition site (Fig. 2a), and BtsCI digested genomic DNA was used as the template. qRT-PCR analysis showed that there were 88-fold and 7-fold more copies of undigested DNA in the *modM3* ON and *modM3* OFF samples, respectively, relative to the ∆*modM3* sample (set as 1 as a reference) (Fig. 2d). Minimal amplification was seen for the ∆*modM3* sample, likely due to incomplete DNA digestion by the temperature sensitive BtsCI. The presence of methylated, undigested sites in the *modM3* OFF population can be attributed to the 15% of cells having phase varied to *modM3* ON, as indicated by GeneScan fragment length analysis (Fig. 1b).

### ModM3 phase variation regulates the expression of multiple genes in a phasevarion

To determine whether phase variation of ModM3 alters expression of a phasevarion, the transcriptomes of *modM3* ON and *modM3* OFF variants were compared using RNASeq analysis. This identified 31 genes that are differentially regulated between the *modM3* ON and *modM3* OFF variants when grown to mid-log phase in aerated culture (≥1.25 fold expression ratio, *P* ≤ 0.05, Table 2). Seventeen genes were up-regulated and fourteen genes down-regulated in *modM3* ON versus *modM3* OFF. The *modM3* gene and downstream *res* gene displayed the greatest difference in expression (9.03 and 5.49 fold increased expression in *modM3* ON versus *modM3* OFF, respectively), and *ddc* gene downstream of *res* also had increased expression (RS065760, 1.27 fold) relative to the *modM3* OFF variants. Four genes involved in the nitrosative stress response (*aniA, norB, narL,* and *narX*), one gene potentially involved in the response to oxidative stress (RS03200), and three genes belonging to a Type I-F CRISPR-cas system operon (*csy1*, *csy2* and *csy3*) were upregulated in *modM3* ON. In addition, two genes (*traW* and RS09480) located on the large unnamed 195ME conjugative plasmid (GenBank accession NZ_CP018060.1 [25]) were also upregulated in *modM3* ON. Three genes that encode part of the NADH:ubiquinone oxidoreductase (*nuoB*, *nuoK* and RS05045) were downregulated in *modM3* ON. These results demonstrate that expression status of ModM3 affects the expression of multiple genes that have diverse functions and subcellular locations (Table 2). This confirms that ModM3 regulates a phasevarion.

**Table 2:** Differentially expressed genes from RNASeq analysis of *M. catarrhalis* 195ME ModM3 ON and OFF strains

Locus tag	Annotation	Fold change ON:OFF	Function (Localization)
Increased expression in *modM3* ON			
RS06580	Type III DNA methyltransferase ModM3	9.03	Restriction/modification (C)
RS06575	Type III restriction endonuclease Res	5.49	Restriction/modification (C)
RS08120	Type I-F CRISPR-associated protein Csy1	1.44	Unknown (C)
RS04785	Nitrate/nitrite response regulator NarL	1.40	Transcription regulation (C)
RS07320	YcgN family cysteine cluster protein	1.39	Unknown (U)
RS08115	Type I-F CRISPR-associated protein Csy2	1.36	Unknown (C)
RS07645	Copper-containing nitrite reductase AniA	1.33	Nitrogen metabolism (OM)
RS09480	Hypothetical protein	1.33	Unknown (U)
RS07655	Nitric oxide reductase NorB	1.32	Nitrogen metabolism (IM)
RS03495	DNA repair protein RadA	1.31	DNA repair (C)
RS08110	Type I-F CRISPR-associated protein Csy3	1.31	Unknown (C)
RS09415	Type-F conjugative transfer system protein TraW	1.29	Conjugation (U)
RS04780	Nitrate/nitrite sensor histidine kinase NarX	1.29	Signal transduction (IM)
RS06570	L-2,4-diaminobutyrate decarboxylase ddc	1.27	Intermediary metabolism (C)
RS03200	AhpC/TSA family peroxiredoxin	1.27	Oxidative stress response (C)
RS00630	2,4-dienoyl-CoA reductase FadH	1.27	Fatty acid metabolism (C)
RS02150	Signal transduction protein	1.27	Unknown (U)
Decreased expression in *modM3* ON			
RS05055	NADH-quinone oxidoreductase subunit NuoB	0.79	Energy metabolism (IM)
RS04595	Hypothetical protein	0.78	Unknown (U)
RS03500	Sulfate ABC transporter ATP-binding protein CysA	0.78	Transport and binding proteins (IM)
RS04010	DUF1049 domain-containing protein	0.77	Unknown (IM)
RS04620	Hypothetical protein	0.76	Unknown (U)
RS06595	7-carboxy-7-deazaguanine synthase QueE	0.75	Nucleotide metabolism (C)
RS05010	NADH-quinone oxidoreductase subunit NuoK	0.74	Energy metabolism (IM)
RS02815	Hypothetical Moraxella Phage protein	0.72	Prophage functions (U)
RS04610	Hypothetical protein	0.70	Unknown (U)
RS09365	Hypothetical protein	0.66	Unknown (U)
RS06515	Putative membrane protein	0.64	Cell envelope (IM)
RS00885	Hypothetical protein	0.63	Unknown (C)
RS06750	Hypothetical protein	0.63	Unknown (U)
RS05045	Sulfite exporter TauE/SafE family protein	0.62	Anion transport (IM)

Gene locus tags and annotations are from *M. catarrhalis* 195ME (NCBI RefSeq accession number NZ_CP018059.1. Genes with ≥1.25 fold change between the ModM3 ON and OFF variants with a *P* value ≤0.05 were included. Function and subcellular location were determined by BLASTp search against the Uniprot database. Subcellular localization was determined by: *C* Cytoplasm, *IM* Inner membrane, *OM* Outer membrane, *U* Unknown

### ModM3 phase variation does not result in phenotypic differences in vitro

To investigate the phenotypic effects of ModM3 phase variation, *M. catarrhalis* ON and OFF variants were compared in in vitro assays, intended to simulate conditions relevant to infection of the human host. Growth curve analysis showed no significant difference in growth rate or final optical density between the ModM3 ON and OFF variants (Additional file 1: Figure S1a). The *modM3* ON and *modM3* OFF variants showed similar levels of survival under conditions of oxidative stress (hydrogen peroxide assays; Additional file 1: Figure S1b), and similar levels biofilm formation over 24, 48 and 72 h (Additional file 1: Figure S1c). Expression of ModM3 also did not significantly affect capacity of *M. catarrhalis* to adhere to or invade A549 cells (Additional file 1: Figure S1d).

### ModM3 expression affects infection dynamics of *M. catarrhalis* and NTHi in the chinchilla model of experimental otitis media

**Fig. 3:**
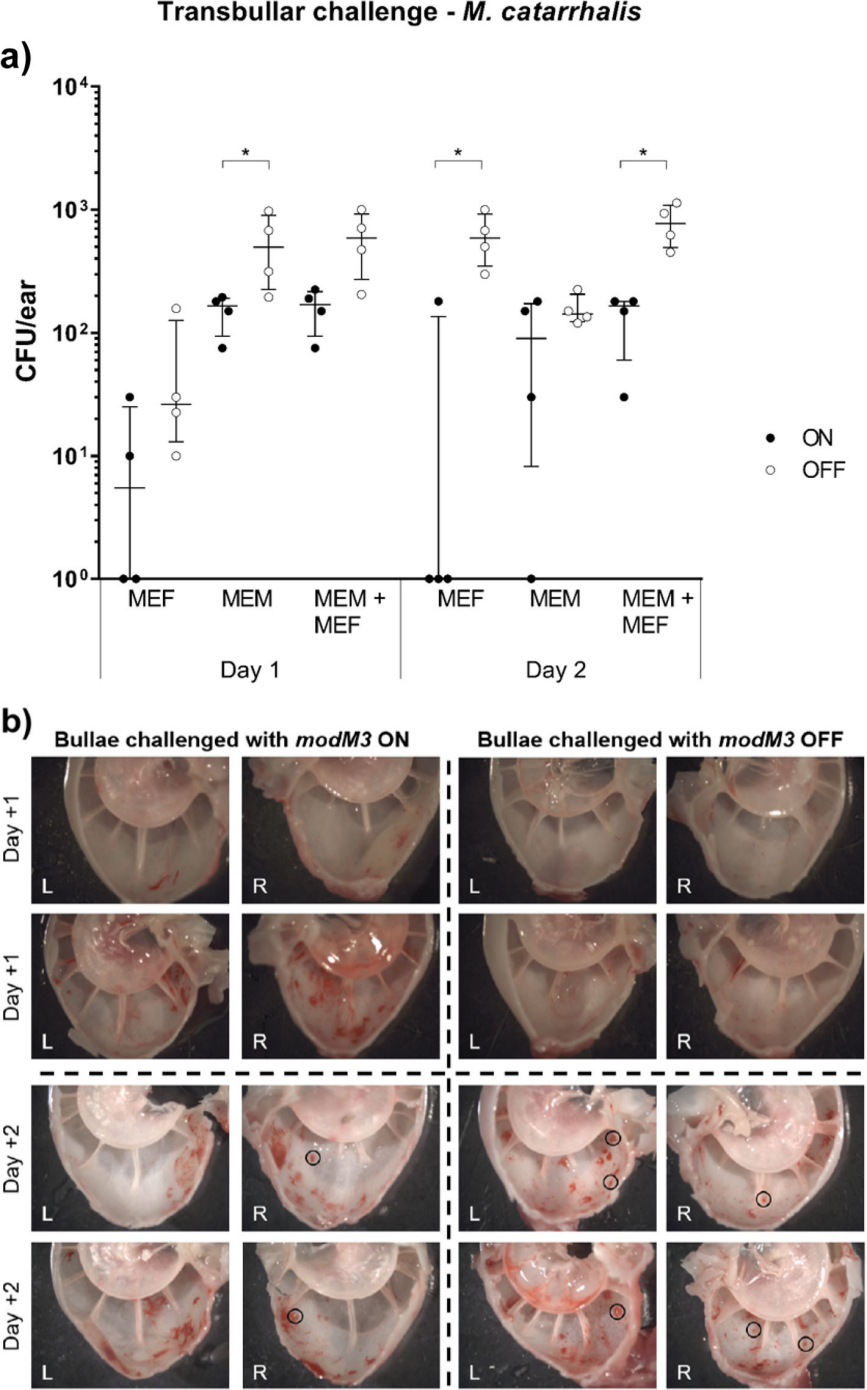
ModM3 expression alters survival of *M. catarrhalis* in chinchilla ears. **a** Number of *M. catarrhalis* CFU found in middle ear fluids (MEF), middle ear mucosa (MEM), or whole bullae (MEF + MEM) from chinchilla ears transbullarly inoculated with either *M. catarrhalis modM3* ON or *modM3* OFF. The median CFU and interquartile range is displayed for each cohort. * indicates *P* < 0.05. **b** Images of middle ears challenged with *M. catarrhalis modM3* ON or *modM3* OFF populations. Submucosal pockets of bacteria, characteristic of OM, are indicated by black circles

We assessed whether differences in gene expression between *M. catarrhalis modM3* ON and *modM3* OFF variants impacts colonisation and/or virulence in vivo, using a chinchilla model of experimental OM [26]. Chinchillas were challenged by transbullar injection with *modM3* ON or *modM3* OFF populations and the number of *M. catarrhalis* colony forming units (CFU) in middle ear fluids and mucosal membrane homogenates were counted at days + 1 and + 2 post challenge. At all time points, a greater number of *M. catarrhalis* CFU were isolated from middle ear fluids and mucosal membranes of ears challenged with the *modM3* OFF input pool than from chinchillas challenged with the *modM3* ON population. This difference was statistically significant for middle ear mucosa samples on day + 1, middle ear fluid samples on day + 2, and total CFU per ear on day + 2 (*P* < 0.05, Mann-Whitney test) (Fig. 3a). Due to differential gene regulation between *modM3* ON vs OFF, bacterial load may not necessarily be linked to disease severity. The severity of disease caused by *M. catarrhalis* phase variants was assessed using tympanometry and imaging of dissected bullae to monitor for signs of OM. On day + 1 post challenge small regions of erythema were observed in the middle ears of chinchillas from both cohorts, with an apparent slight increase in the ears challenged with the *modM3* ON population (Fig. 3b). At Day + 2 post challenge, obvious erythema was observed in all middle ears with no clear difference seen in the relative amount of erythema present between cohorts. However, the development of a greater number of submucosal pockets of bacteria was observed in bullae of chinchillas challenged with *modM3* OFF compared to *modM3* ON (Fig. 3b). Middle ear pressure reduction is characteristic of Eustachian tube dysfunction in OM which hinders equilibration of the middle ear space against barometric pressure in the nasopharynx. The mean middle ear pressure on day + 1 and day + 2 post challenge was below the normal range for chinchillas (+/− 60 daPa), for both *modM3* ON and *modM3* OFF challenged cohorts, consistent with infection. A lower middle ear pressure was observed in chinchillas challenged with *modM3* OFF compared to the cohort challenged with *modM3* ON at day + 1 and day + 2 post challenge, however this difference was not significant (*P* > 0.05) (Additional file 1: Figure S2a). Expression of ModM did not affect tympanic membrane compliance, as values were within the normal range for chinchillas (0.75 to 1.5) and not significantly different between cohorts (*P* > 0.05) (Additional file 1: Figure S2b).

**Fig. 4:**
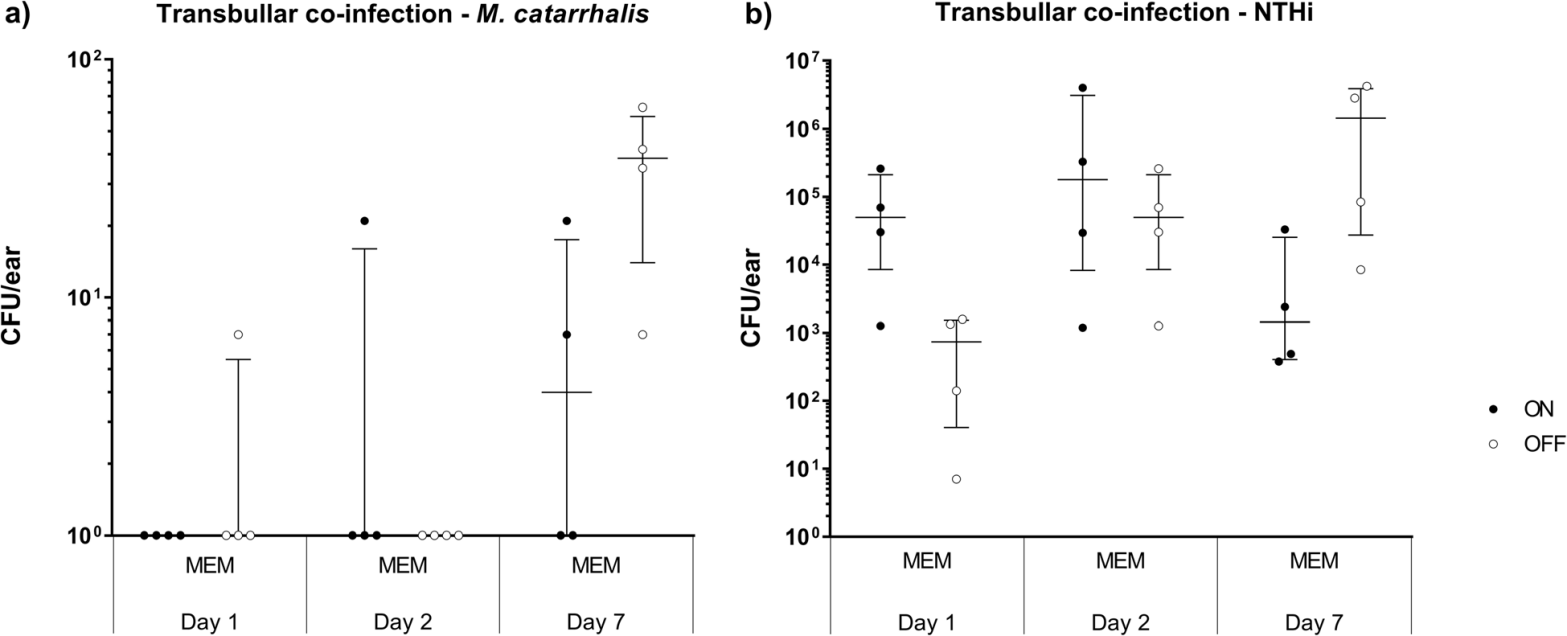
ModM3 expression alters survival of NTHi in chinchilla ears. **a** *M. catarrhalis* and **b** NTHi CFU in MEM samples from chinchilla bullae co-challenged with *M. catarrhalis modM3* ON or *modM3* OFF, plus NTHi. The median CFU is and interquartile range is displayed for each cohort

In order to determine whether *modM3* phase variation also affects infections in polymicrobial challenge, chinchillas were co-challenged with NTHi strain 86-028NP (which contains a *modA2* genes that only contains three DNA repeats and is therefore not phase variable) in combination with either *M. catarrhalis modM3* ON, or *modM3* OFF populations. Middle ear fluid and mucosal membrane homogenates were counted at days + 1, + 2 and + 7 post challenge. At days + 1 and + 2 post challenge the number of *M. catarrhalis* CFU was below the level of detection in the majority of mucosal membrane homogenates. However, *M. catarrhalis* was detected in mucosal membrane homogenates at day + 7 in 2/4 animals challenged with *modM3* ON versus 4/4 animals challenged with *modM3* OFF (Fig. 4a). Although more NTHi was recovered from mucosal membrane homogenates of ears co-challenged with *M. catarrhalis modM3* ON at days + 1 and + 2 post challenge, and a greater number of NTHi CFU were retrieved from ears co-challenged with *M. catarrhalis modM3* OFF on day + 7, these differences were not significant (Fig. 4b). *M. catarrhalis* and NTHi were not detected in middle ear fluids at any time point.

## Discussion

*M. catarrhalis* is a leading bacterial pathogen of OM in children and exacerbations of COPD in adults. Despite the significant morbidity caused, factors contributing to *M. catarrhalis* virulence and their role in the progression of OM and exacerbations of COPD have not been fully elucidated, and there is currently no vaccine available to protect against *M. catarrhalis* infection. The presence of phasevarions in bacterial pathogens results in increased phenotypic diversity of these organisms. Where multiple allelic variants of phase-variable methyltransferases are present, each unique Mod allelic variant has been shown to methylate a unique target sequence, and regulate a different phasevarion [17, 21, 27]. Six *modM* alleles (*modM1–6*) have been identified in *M. catarrhalis* that vary in their central target recognition domain, and are therefore all expected to regulate distinct phasevarions [22]. Our previous analysis showed that ModM2 methylates the target sequence 5′-GAR^m6^AC-3′ and regulates a phasevarion containing 34 genes [23]. In this study we have characterized the ModM3 phasevarion in *M. catarrhalis* strain 195ME, showing that phase variation of ModM3 is mediated by changes in the number of 5′-(CAAC)_n_-3′ repeat units present in the repeat tract of the *modM3* ORF, and that the ModM3 protein methylates the sequence 5′-AC^m6^ATC-3′.

We demonstrated expression of a suite of genes differs in populations of ModM3 ON and OFF. Under aerobic growth, to mid-log phase, 29 genes were differentially expressed (1.27–1.61 fold). Five genes involved in the response to oxidative and nitrosative stress are upregulated in *modM3* ON versus *modM3* OFF: the nitrite and nitric oxide reductases *aniA* and *norB,* the two-component system *narX/narL,* and the predicted AhpC/TSA family peroxiredoxin RS03200. AniA and NorB are components of the truncated *M. catarrhalis* denitrification pathway required for survival in the presence of exogenously generated reactive nitrogen species [28, 29]. Detoxification of nitric oxide by NorB in *N. meningitidis* was shown to enhance intracellular survival within macrophages and nasopharyngeal mucosal cells [30]. Whether increased expression of NorB in *modM3* ON confers greater protection against macrophage-generated nitric oxide warrants further investigation. Gene expression experiments [31] found that *aniA* and *norB* are highly upregulated during growth in biofilms versus planktonic growth in vitro, suggesting a role for both genes in the progression of OM as *M. catarrhalis* biofilms have been observed in middle ears of children with chronic OM [32]. However, we observed no difference in biofilm formation between our *modM3* ON and *modM3* OFF strain pair, suggesting that the role of ModM3 in regulation of *aniA* and *norB* is more subtle, and may only occur under specific in vivo conditions that we are unable to reproduce in the in vitro assay. The *narX* and *narL* operon form a two-component regulatory system that responds to nitrate and nitrite stimuli and modulates gene expression. The genes regulated by NarX-NarL have not been defined in *M. catarrhalis*, however in other organisms NarX-NarL induces expression of genes important for survival and growth in anaerobic environments [33, 34]. The middle ear is a microaerophilic environment [35] and anaerobic conditions may be reached in mature biofilms [36]. Upregulation of NarX/L in the *modM3* ON strain may promote persistence in the middle ear through increased survival within biofilms under anaerobic conditions, where additional regulators may be induced and be acting in concert with ModM3. Intriguingly, several of these genes, or pathways, have previously been noted to be upregulated in *M. catarrhalis* when isolated from the nasopharynx of chinchillas, including *aniA*, *norB*, and other genes associated with denitrification pathway, while in the chinchilla nasopharynx, elements of energy production (NADH:ubiquinone oxidoreductases) are down regulated [37]. These in vivo expression changes have parallels to the expression changes we noted in aerobic, log phase *modM3* ON strains, suggesting this population may have a pre-adapted advantage to in vivo growth in the nasopharynx. Although expression of the peroxiredoxin AhpC was increased 1.27 fold in our ModM3 ON strain, we observed no difference in survival between our *modM3* ON and OFF variants following exposure to hydrogen peroxide. It is possible that the observed difference in AhpC expression was too small to have a noticeable impact on CFU counts in this assay. Alternatively, as AhpC is only minimally involved in H_2_O_2_ degradation in *M. catarrhalis* compared to catalase (KatA) [38], the effect of differential regulation of AhpC may have been undetectable in this assay. The lack of detectable in vitro phenotypic differences between the *modM3* ON and OFF strains may be due to the narrow range of conditions able to be replicated in vitro compared to in vivo. Our gene expression analysis was also only carried out under aerobic culture conditions, and thus the present characterization may not encompass the entire suite of genes that are differentially regulated by ModM3 phase variation. In order to observe the differential regulation of genes that are not constitutively expressed, further characterization experiments need to be performed with cells grown in vivo or under a range of in vitro conditions that simulate the physiological environment encountered upon infection of the human host and where various regulatory proteins mediated expression. DNA methylation typically affects DNA interactions of regulatory proteins involved in transcription, rather than acting directly on transcriptional machinery (reviewed in [39]). As such, ModM3 may impact genes expression either directly by methylation changes at the promoter region of the regulated gene that affect interactions with active regulators, or indirectly by altering expression of regulatory proteins [40].

We have previously examined the role of phasevarions in animal models using other causative agents of OM: it was noted that the *modA2* ON state was selected in NTHi in chinchilla [21, 41]. This indicated that a particular expression state of a phase-variable methyltransferase, and the genes they regulate, gives an advantage in this anatomical niche. We investigated *M. catarrhalis* infection dynamics using a chinchilla infection model and observed differences between the *M. catarrhalis modM3* ON and *modM3* OFF strain variants despite not detecting any in vitro phenotypic differences between this strain pair. Expression profiles of our aerobically grown *modM3* ON more closely resembled the previously reported expression profile of *M. catarrhalis* isolated from chinchilla nasopharynx [37], including changes in oxidative stress, and denitrification pathways. Given this, it was a surprise that infection with *M. catarrhalis* strain 195ME *modM3* ON resulted in a decreased bacterial load in chinchilla ears compared to *modM3* OFF infected animals, as we predicted the *modM3* ON mediated phasevarion would provide a survival advantage. This highlights that the nasopharynx and middle ear are distinct compartments presenting different challenges and selective pressures, and that in vivo models reflect the complexity of human carriage and disease.

The chinchilla model of OM has also been used to study polymicrobial interactions between the three major bacterial OM pathogens: *M. catarrhalis* survival increased when co-infected with *H. influenzae* compared to infection with *M. catarrhalis* alone [42]. The impact of switching of expression of the ModM3 phasevarion on survival in polymicrobial OM was investigated by co-infecting chinchillas transbullarly with either *M. catarrhalis modM3* ON or *modM3* OFF variants in combination with NTHi. Although the observed difference of *M. catarrhalis* and NTHi CFU retrieved from bullar mucosal membrane homogenates was not statistically significant, it would seem that co-infection with NTHi reduced the level of infection by *M. catarrhalis*, compared to when the animals were challenged with *M. catarrhalis* alone*.* However, co-infection with the *M. catarrhalis modM3* ON variant provided an advantage to survival to NTHi during early infection (days + 1 and + 2), while *modM3* OFF enhances survival of NTHi at later stages of OM. Phenotypic analysis of bacteria that contain several phase-variable genes, such as *M. catarrhalis*, can be confounded by phase variation of genes that are not the subject of investigation. It is unlikely that genes other than *modM3* have phase varied during isolation of the ModM3 ON and OFF populations, as none of the genes identified as differentially regulated by RNASeq are known to be phase variable. However, the low bacterial recovery from in vivo chinchilla experiments prevented analysis of the expression status of other known phase variable genes (i.e., *uspA1*, *uspA2*, and *mid/hag*) and we cannot definitively say that the phenotypes seen in the animal model were solely due to the ON vs OFF status of ModM3. Furthermore, we were also unable to determine whether there was selection of ModM3 ON or OFF variants during in vivo infection. It is important to note that there are several limitations to the animal model data presented due to the small number of animals tested in addition to the low numbers of bacteria recovered in each experiment. Although the chinchilla model is considered the gold standard for investigating OM [43], *M. catarrhalis* is a human-host adapted bacteria that does not naturally infect any known animal model. Therefore, investigations in animals are challenging and further experimentation is required to validate these in vivo observations, ideally from samples collected from humans with OM.

## Conclusions

In this study we have confirmed that ModM3 is phase variable, identified the ModM3 methylation target sequence, and characterized the ModM3 phasevarion. ModM3 regulates the expression of multiple genes (15 gene upregulated and 14 genes downregulated 1.27–1.61 fold between the ModM3 ON vs OFF states), belonging to pathways important for infection of the human host, including those involved in biofilm formation and in the response to oxidative and nitrosative stress. Our observations that the *modM3* OFF state may confer a survival advantage to *M. catarrhalis* in vivo, and that phase variation of *modM3* may alter survival of NTHi at different stages of infection has important implications in OM progression and treatment. The observed modulation of gene expression may facilitate adaptation of *M. catarrhalis* to changing host microenvironments, potentially promoting persistence of *M. catarrhalis* in the middle ear and contributing to the ability of *M. catarrhalis* to cause OM.

## Methods

### Bacterial strains and growth conditions

*M. catarrhalis* strains used in this study include American Type Culture Collection (ATCC) 25,239 [23] and CCRI-195ME, an OM isolate recovered from the middle ear of a 16-month-old otitis-prone child, at the Columbus Children’s Research Institute (CCRI), Nationwide Children’s Hospital, Columbus, Ohio [25]. *M. catarrhalis* strains were routinely cultured on brain heart infusion (BHI) agar (Oxoid, Basingstoke, UK) at 37 °C with 5% CO_2_ overnight (~ 16 h). For use in all biological assays, cells cultured overnight on BHI agar were re-suspended in 20 mL of BHI broth (Oxoid, Basingstoke, UK) to an optical density at 600 nm (OD_600_) = 0.05 and grown for an additional 4 h to mid log phase at 37 °C with orbital shaking at 200 rpm.

### Construction of the *M. catarrhalis modM3* knockout mutant strain

To generate the 195ME *modM3*-knockout mutant (*∆modM3*), 195ME was transformed with 1 μg of BsaI linearized plasmid pMcrepI::*kan* [23] by co-incubation on BHI agar at 37 °C, 5% CO_2_ for 3 h. Transformants were selected on BHI agar containing 20 μg/mL kanamycin and confirmed by PCR analysis.

### Fragment length analysis

The length of the *modM* 5′-(CAAC)_n_-3′ tetranucleotide repeat tract in *M. catarrhalis* 195ME, and the proportion of each fragment length present was determined using GeneScan fragment analysis as per [23]. Briefly, the *modM* repeat region was amplified with the 6-carboxyfluorescein (6-FAM) labelled forward primer Mcmod2F-6FAM (5′-[6FAM]-TTACTTGACACTCTGAATGGA-3′) and the unlabelled reverse primer McmodrepR2 (5′-GTATTATGGGCAGTTTTTAAG-3′) using GoTaq Flexi DNA polymerase (Promega) in 20 μL reaction volumes as per manufacturer’s instructions. Analysis of fluorescent traces and quantification of fragment lengths was performed using PeakScanner v1.0 (Applied Biosystems, Grand Island, NY, USA). *M. catarrhalis* subpopulations containing different *modM3* repeat tract lengths were selected by fragment length analysis of individual colonies, as previously described [20].

### Western blot analysis

*M. catarrhalis* 195ME *modM3* ON, *modM3* OFF were grown to mid log phase as described above. Cultures were then centrifuged at 6000 x *g* for 5 min to pellet, and the pellets were re-suspended in water to an OD_600_ of 5. Cell lysates were prepared by boiling for 10 min and 20 μg of protein was electrophoresed on a 4–12% Bis-Tris NuPAGE polyacrylamide gel at 180 V for 60 min. Protein was transferred to a nitrocellulose membrane using the XCell II Blot Module at 30 V for 70 min. Western blot analysis was performed using a mouse polyclonal anti-Mod antibody at 1:1000 dilution, followed by a 1:5000 dilution of a rabbit anti-mouse alkaline phosphatase conjugated antibody. Western blots were developed using the AP Conjugate Substrate Kit (Bio-Rad) as per manufacturer’s instructions.

### Methylome determination by single molecule real-time (SMRT) sequencing

The 195ME *modM3* ON (36 repeats), *modM3* OFF (37 repeats), and ∆*modM3* strains were grown to mid-log phase as described above. Genomic DNA was extracted using the Qiagen Genomic-tip 20/G kit (Qiagen) and SMRTbell libraries were prepared as described previously [23, 44]. Sequencing was performed on the PacBio RS II (Menlo Park, CA, USA) using standard protocols for short and long insert libraries.

### Confirmation of the ModM3 methylation target sequence

#### Restriction inhibition assay

Genomic DNA was extracted from mid log cultures of 195ME *modM3* ON, *modM3* OFF, and ∆*modM3* strains using GenElute Bacterial Genomic DNA Kit (Sigma-Aldrich). Genomic DNA was digested with the restriction enzyme BtsCI (NEB) at 50 °C or HindIII (NEB) at 37 °C for 60 min. Restriction enzymes were heat inactivated at 80 °C for 20 min. Digested and undigested genomic DNA was electrophoresed on a 0.8% agarose gel at 110 V for 50 min.

#### Southern blotting

Electrophoresis of BtsCI digested DNA performed as above and was then transferred to a charged nylon membrane by the capillary transfer method [45]. The DNA probe for Southern hybridization was amplified using the primer pair M3ResInh-F (5′- TTTAGCAAAGGGTAACCACC-3′) and M3ResInh-R (5′- ACAATGACACGCTCAACTCG-3′) and labelled with Digoxigenin (DIG) using the DIG-High Prime DNA Labelling and Detection Starter Kit II (Roche) as directed by the manufacturer.

#### Quantitative real-time PCR

Quantitative real-time PCR was performed using Sybr Green Master Mix (Bio-Rad) in 20 μL reaction volumes with 1 ng of BtsCI digested genomic DNA as template and 250 nmol of each primer. qRT-PCR was performed on the CFX96 Touch™ Real-Time PCR Detection System (Bio-Rad) with the following cycle conditions: polymerase activation at 98 °C for 2 min, followed by 40 cycles of denaturation at 98 °C for 15 s, and annealing/extension at 60 °C for 30 s. The primers M3Met_RT_F (5′-CAGGTTCGCCATCAACAAAC-3′) and M3Met_RT_R (5′-TGCCTGCCATCGCTGAATC-3′) were designed to amplify a 99 bp amplicon spanning an overlapping BstCI and ModM3 recognition sequence. The primers CopB_RT-F (5′- GTGAGTGCCGCTTTTACAACC-3′) and CopB_RT-R (5′- TGTATCACCTGCCAAGACAA − 3′) were used in control reactions and amplify a 72 bp amplicon in the *copB* gene that does not contain a BtsCI cleavage site. Relative quantitation of BtsCI digested sites in each sample was performed using the ∆∆Ct method.

### RNA sequencing

*M. catarrhalis* strains 195ME ModM3 ON and OFF were cultured in triplicate on BHI agar at 37 °C with 5% CO_2_ overnight (16 h). Each strain was then re-suspended in 20 mL BHI broth to an optical density of OD_600_ = 0.05 and grown for a further 3 h at 37 °C with orbital shaking at 200 rpm. 2 mL of culture was added directly to 4 mL of RNAprotect Bacteria Reagent (Qiagen), and incubated at room temperature for 5 min. Cultures were pelleted by centrifugation at 5000 x *g* for 10 min at 4 °C and the supernatant was discarded. Pellets were resuspended in 200 μL of lysis buffer (30 mM Tris·Cl, 1 mM EDTA, pH 8.0) containing 15 mg/mL lysozyme and 80 units/mL proteinase K. Total RNA was extracted using RNeasy Mini Kit (Qiagen) and residual DNA digested using RNase-Free DNase Set (Qiagen) as per manufacturer’s instructions. RNASeq was performed by the Australian Genome Research Facility (Melbourne, VIC, Australia). RNA libraries were prepared using the Illumina Ribo-Zero Gold protocol. Pooling and clustering of libraries was performed using the Illumina cBot system with TruSeq PE Cluster kit v3 reagents. Sequencing (150 bp paired-end reads) was performed on the Illumina MiSeq system with TruSeq SBS Kit v3 reagents.

### Growth curve analysis

*M. catarrhalis* overnight plate cultures were re-suspended in 1 mL BHI broth and equalized to an OD_600_ of 0.05 and 100 μL volumes of each bacterial suspension was transferred to a 96 well plate in triplicate. Automated growth curve analysis was performed using a Tecan Infinite M1000 Pro microplate reader. Growth curves were performed at 37 °C with orbital shaking at an amplitude of 4 mm, and spectroscopy readings were taken at a wavelength of 600 nm at 1 h intervals.

### H_2_O_2_ killing assay

*M. catarrhalis* strains were grown to mid log phase as described above. Mid-log phase cultures were diluted in BHI broth to 1 × 10^6^ CFU/mL and 90 μL of each suspension was added to wells of a 96 well plate in triplicate. The killing assay was started by addition of 10 μL H_2_O_2_ in BHI broth to a final concentration of either 5 or 10 mM H_2_O_2_ and plates were incubated at 37 °C, 5% CO_2_. Samples were taken at 15, 30, 45 and 60 min, 10 fold serial dilutions were performed in BHI broth containing catalase, and 5 μL of each dilution was plated onto BHI agar and incubated overnight at 37 °C, 5% CO_2_.

### Biofilm formation assay

*M*. *catarrhalis* biofilm assays were performed as previously described [46]. Briefly, strains were standardized to OD_600_ = 1.0 in BHI broth and diluted 1:10 in chemically defined media (final OD_600_ = 0.1). Biofilm assays were performed in 0.5 mL volumes in 48-well tissue culture plates, and grown without agitation for 24, 48, or 72 h at 37 °C, 5% CO_2_. Wells were stained with 0.05% crystal violet (Sigma-Aldrich) for 15 mins, washed three times with PBS, and crystal violet was solubilised with 90% ethanol. Quantification was performed at 600 nm using the Tecan Infinite M1000 Pro microplate reader.

### Adherence and invasion assays

The A549 human lung epithelial cell line (ATCC, CCL­185) was maintained in Minimal Essential Media (MEM) supplemented with 10% Foetal Bovine Serum at 37 °C, 5% CO_2_. A549 cells were grown to confluent monolayers in 24-well tissue culture plates (1 × 10^5^ cells/well) and infected with 1 × 10^6^ *M. catarrhalis* 195ME cells. Plates were incubated for 3 h at 37 °C, 5% CO_2_. For adherence assays, non-adherent bacteria were removed by washing the wells three times with 1 mL PBS. For invasion assays, extracellular bacteria were removed by treating wells with 500 μL of 100 μg/mL gentamycin for 15 min at 37 °C, 5% CO_2_ and then washing with PBS as above. Cells were detached from the plate by treatment with 200 μL of 0.25% trypsin for 10 min at 37 °C, 5% CO_2_, and lysed by adding 300 μL of 1% saponin and thorough vortexing. To enumerate adherent and intracellular bacteria, 10 fold serial dilutions of each sample were performed in BHI broth and 5 μL of each dilution was plated onto BHI agar and incubated overnight at 37 °C, 5% CO_2_.

### Chinchilla model of OM

Due to anatomical similarities of the chinchilla (*Chinchilla lanigera)* and human middle ear, and immunological similarities with children in the response to middle ear infection, the chinchilla model has been extensively used to study prominent otopathogens, including *M. catarrhalis*, in the pathogenesis of OM [43].

#### Housing of chinchillas and experimental procedures

Adult, outbred, chinchillas of mixed sex were acquired from Rausher Chinchilla Ranch (LaRue, Ohio) and allowed to acclimate to the vivarium for 7 days prior to the beginning of the study. Animals were housed individually in clear cages with autoclaved corn cobb bedding and access to autoclaved water and certified feed ad libitum. Racks of cages were maintained in negative air flow isolation units with a 12 h light cycle. All animals were examined by a veterinarian upon arrival for general health and were examined twice each day by veterinary staff and laboratory personnel in accordance with Institutional Animal Care and Use Committee (IACUC) guidelines. No deviations were observed in health or behaviour over the course of the study. Cohort sizes for each study were determined based on extensive prior experience with this animal model and in keeping with the principles of Replacement, Refinement and Reduction in animal numbers. Animals were randomly clustered into cohorts such that the mean weight of each cohort was comparable. Animals were examined in numerical order based on their ear tag, not by cohort clustering. Based on American Veterinary Association (AVMA) Guidelines for Euthanasia of Animals, the primary method for euthanasia was cardiac injection of Euthosol (10 mg ketamine-HCl plus 2 mg xylazine per kg body weight) to anesthetized animals followed by decapitation as the secondary physical method for euthanasia.

#### Transbullar infection of M. catarrhalis

Eight chinchillas were weighed and divided into two cohorts of four animals with equal mean weight (609 +/− 76 g). On Day + 0, cohorts were challenged with either *M. catarrhalis* 195ME *modM3* ON or *modM3* OFF variants. Challenge doses consisted of 2800 CFU in 300 μL of saline delivered directly into both the left and right bulla for each animal. Tympanometry was performed on a daily basis to assess progression of otitis media. On day + 1 and day + 2 post challenge, two animals from each cohort were sacrificed, and the bullae were dissected from the skull and aseptically opened and imaged. CFU enumeration was performed by plating dilutions of middle ear effusates and mucosal membrane homogenates on chocolate agar.

#### Transbullar co-infection of M. catarrhalis and NTHi

Twelve chinchillas were divided into two cohorts of six animals with equal mean weight (663 +/− 21 g). On Day + 0 cohorts were challenged with NTHi strain 86-028NP in combination with either *M. catarrhalis* 195ME *modM3* ON or *modM3* OFF variants. Transbullar challenge was performed as previously stated, with challenge doses consisting of 1000 CFU for NTHi and 3000 CFU for *M. catarrhalis* being co-administered into each bulla. Two animals from each cohort were sacrificed on days + 1, + 2, and + 7 post challenge and the bullae were dissected from the skull and aseptically opened and imaged. In order to discriminate between *M. catarrhalis* and NTHi and permit enumeration of each bacterial species, dilutions of middle ear effusates and mucosal membrane homogenates were plated on chocolate agar to quantitate NTHi, and chocolate agar with 10 μg/mL vancomycin and 5 μg/mL trimethoprim to select for and quantitate *M. catarrhalis*, as previously reported [47, 48].

#### Statistical analysis

Each chinchilla ear was counted as a separate experimental unit. Exact *P* values were calculated with the Mann-Whitney U test using GraphPad Prism version 7.02.

## Supplementary information

**Additional file 1: Figure S1.** In vitro phenotypic assays of *M. catarrhalis* 195ME *modM3* ON and *modM3* OFF variants. **a**) Automated microtiter plate growth curve analysis of optical density at 600 nm (OD_600_) over 10 h; **b**) hydrogen peroxide (H_2_O_2_) killing assay showing colony forming units/ml (CFU/ml) after exposure to 0, 5 or 10 mM (H_2_O_2_) over 60 min; **c**) biofilm formation assay with biofilm biomass quantified after 24, 48 or 72 h by analysis of OD_600_ after crystal violet staining; **d**) adherence and invasion of A549 human lung epithelial cells assay measures as CFU/ml. For all assays, the mean of three replicates is shown and error bars indicate +/− 1 standard deviation of the mean. **Figure S2.** Tympanometry and imaging of bullae in the chinchilla model of otitis media. **a**) Mean middle ear pressure measured as decapascals (daPa); and **b**) mean tympanic membrane compliance measured as middle ear volume (mL) in two cohorts of chinchillas challenged with either *M. catarrhalis modM3* ON or *modM3* OFF populations. The mean of four ears is shown and error bars indicate +/− 1 standard deviation of the mean.

## Data Availability

The datasets used and/or analysed during the current study are available from the corresponding author on reasonable request. All supplementary material is available online at BMC Genomics. The CCRI-195ME sequence is available in GenBank under the accession number CP018059. The RNA-seq data discussed in this publication have been deposited in NCBI’s Gene Expression Omnibus and are accessible through GEO accession number GSE140417.

## Abbreviations

BHI: Brain Heart Infusion
CFU: Colony forming units
COPD: Chronic obstructive pulmonary disease
NTHi: Non-typeable *Haemophilus influenzae*
OM: Otitis media
qRT-PCR: Quantitative real time PCR
