# Supplementary material for: The Moraxella catarrhalis phase-variable DNA methyltransferase ModM3 is an epigenetic regulator that affects bacterial survival in an in vivo model of otitis media
Source: BMC Microbiol. 2019 Dec 9;19:276. doi: 10.1186/s12866-019-1660-y (PMC6902483; doi:10.1186/s12866-019-1660-y)
Supplement: Supplementary file 1 — Additional file 1: Figure S1. In vitro phenotypic assays of M. catarrhalis 195ME modM3 ON and modM3 OFF variants. a) Automated microtiter plate growth curve analysis of optical density at 600 nm (OD600) over 10 h; b) hydrogen peroxide (H2O2) killing assay showing colony forming units/ml (CFU/ml) after exposure to 0, 5 or 10 mM (H2O2) over 60 min; c) biofilm formation assay with biofilm biomass quantified after 24, 48 or 72 h by analysis of OD600 after crystal violet staining; d) adherence and invasion of A549 human lung epithelial cells assay measures as CFU/ml. For all assays, the mean of three replicates is shown and error bars indicate +/− 1 standard deviation of the mean. Figure S2. Tympanometry and imaging of bullae in the chinchilla model of otitis media. a) Mean middle ear pressure measured as decapascals (daPa); and b) mean tympanic membrane compliance measured as middle ear volume (mL) in two cohorts of chinchillas challenged with either M. catarrhalis modM3 ON or modM3 OFF populations. The mean of four ears is shown and error bars indicate +/− 1 standard deviation of the mean. [file 12866_2019_1660_MOESM1_ESM.docx]

**Supplementary data**

**The *Moraxella catarrhalis* phase-variable DNA methyltransferase ModM3 is an epigenetic regulator that affects bacterial survival in an *in vivo* model of otitis media**

Luke V. Blakeway^1^, Aimee Tan^1^, Joeseph A. Jurcisek^2^, Lauren O. Bakaletz^2^, John M. Atack^1^, Ian R. Peak^1,3^ and Kate L. Seib^1,^*

^1^ Institute for Glycomics, Griffith University, Gold Coast, Queensland, 4215, Australia

^2^ Center for Microbial Pathogenesis, The Research Institute at Nationwide Children's Hospital, Columbus, Ohio, 43215, USA

^3^ School of Medical Science, Griffith University, Gold Coast, Queensland, 4215, Australia

**
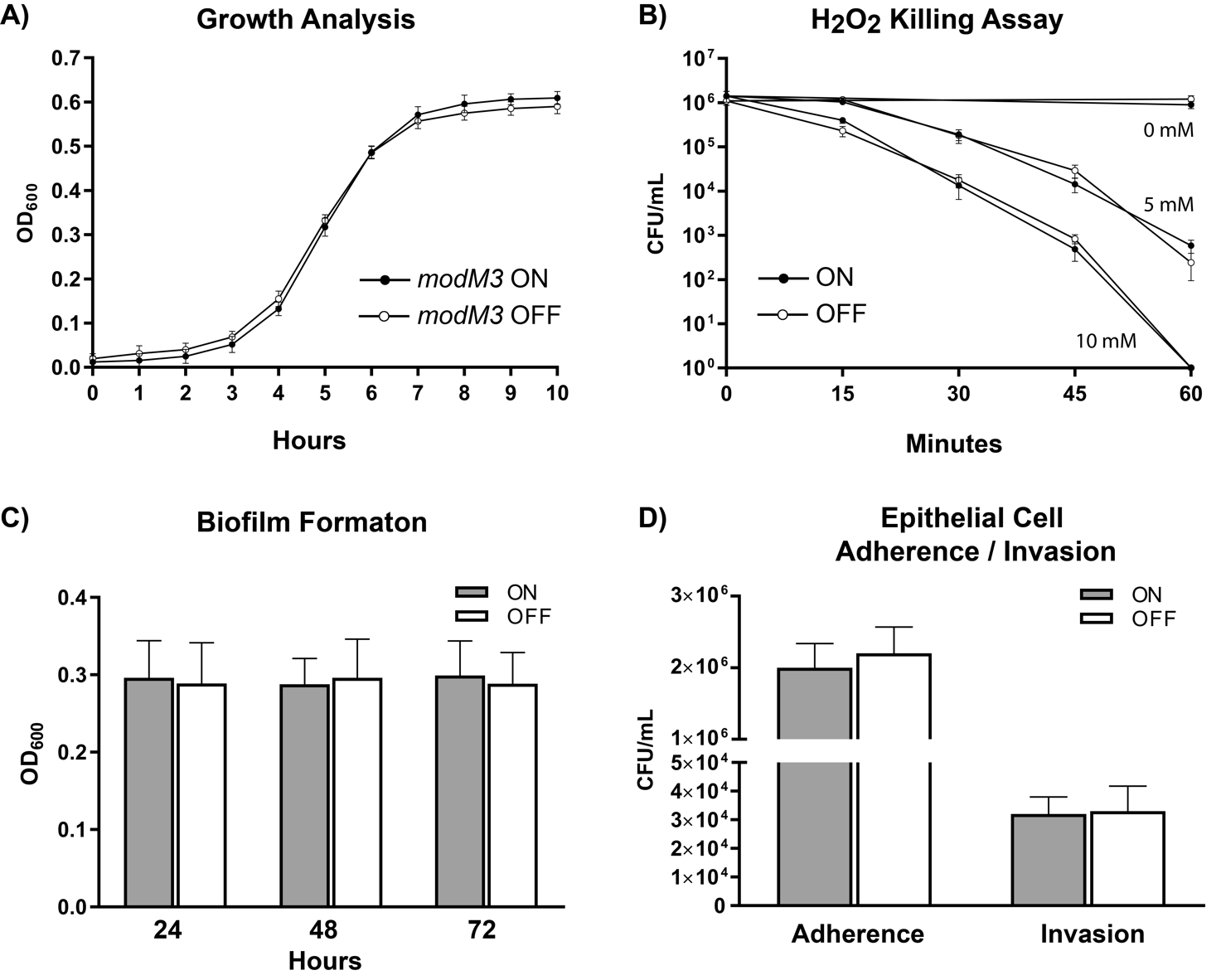
**

**Supplementary Figure 1: *In vitro* phenotypic assays of *M. catarrhalis* 195ME** ***modM3* ON and *modM3* OFF variants. a)** Automated microtiter plate growth curve analysis of optical density at 600 nm (OD_600_) over 10 hours; **b)** hydrogen peroxide (H_2_O_2_) killing assay showing colony forming units/ml (CFU/ml) after exposure to 0, 5 or 10 mM (H_2_O_2_) over 60 minutes; **c)** biofilm formation assay with biofilm biomass quantified after 24, 48 or 72 hours by analysis of OD_600_ after crystal violet staining; **d)** adherence and invasion of A549 human lung epithelial cells assay measures as CFU/ml. For all assays, the mean of three replicates is shown and error bars indicate +/- 1 standard deviation of the mean.

**
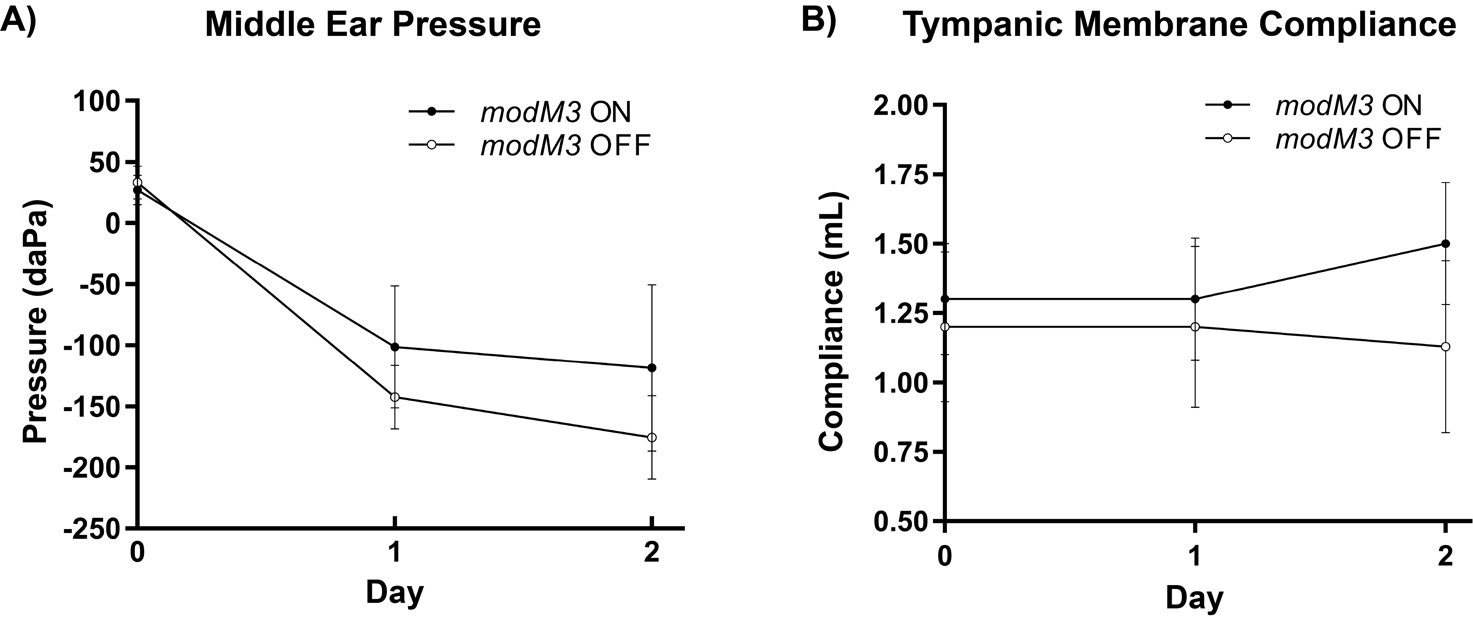
**

**Supplementary Figure 2: Tympanometry and imaging of bullae in the chinchilla model of otitis media. a)** Mean middle ear pressure measured as decapascals (daPa); and **b)** mean tympanic membrane compliance measured as middle ear volume (mL) in two cohorts of chinchillas challenged with either *M. catarrhalis modM3* ON or *modM3* OFF populations. The mean of four ears is shown and error bars indicate +/- 1 standard deviation of the mean.
